# Donor lymphocyte infusions after first allogeneic hematopoietic stem-cell transplantation in adults with acute myeloid leukemia: a single-center landmark analysis

**DOI:** 10.1007/s00277-021-04494-z

**Published:** 2021-04-01

**Authors:** Andrés R. Rettig, Gabriele Ihorst, Hartmut Bertz, Michael Lübbert, Reinhard Marks, Miguel Waterhouse, Ralph Wäsch, Robert Zeiser, Justus Duyster, Jürgen Finke

**Affiliations:** 1 Department of Medicine I, Medical Center - University of Freiburg, Faculty of Medicine, University of Freiburg, Freiburg, Germany; 2 Clinical Trials Unit, University Medical Center Freiburg, Freiburg, Germany

**Keywords:** AML, DLI, Survival, Allo-HSCT, Chimerism, MRD

## Abstract

Allogeneic hematopoietic stem cell transplantation (allo-HSCT) is potentially curative for acute myeloid leukemia (AML). The inherent graft-versus-leukemia activity (GvL) may be optimized by donor lymphocyte infusions (DLI). Here we present our single-center experience of DLI use patterns and effectiveness, based on 342 consecutive adult patients receiving a first allo-HSCT for AML between 2009 and 2017. The median age at transplantation was 57 years (range 19–79), and the pre-transplant status was active disease in 58% and complete remission (CR) in 42% of cases. In a combined landmark analysis, patients in CR on day +30 and alive on day +100 were included. In this cohort (*n*=292), 93 patients received cryopreserved aliquots of peripheral blood-derived grafts for DLI (32%) and median survival was 55.7 months (2-year/5-year probability: 62%/49%). Median survival for patients receiving a first dose of DLI “preemptively,” in the absence of relapse and guided by risk marker monitoring (preDLI; *n*=42), or only after hematological relapse (relDLI; *n*=51) was 40.9 months (2-year/5-year: 64%/43%) vs 10.4 months (2-year/5-year: 26%/10%), respectively. Survival was inferior when preDLI was initiated at a time of genetic risk marker detection vs mixed chimerism or clinical risk only. Time to first-dose preDLI vs time to first-dose relDLI was similar, suggesting that early warning and intrinsically lower dynamics of AML recurrence may contribute to effectiveness of preDLI-modified GvL activity. Future refinements of the preemptive DLI concept will benefit from collaborative efforts to diagnose measurable residual disease more reliably across the heterogeneous genomic spectrum of AML.

**Supplementary Information:**

The online version contains supplementary material available at 10.1007/s00277-021-04494-z.

## Introduction

Allogeneic hematopoietic stem cell transplantation (allo-HSCT) is an effective, potentially curative treatment in patients with a range of acute and chronic leukemias, based on a potent graft-versus-leukemia (GvL) immune effect [1]. Moreover, when leukemic cells recur after allo-HSCT, single or repeated infusion of lymphocytes from the original donor in the absence of prophylactic immune suppression can lead to durable remissions. The flexibility of using donor lymphocyte infusions (DLI) has increased with the finding that cryopreserved aliquots of the donor cell preparation used for the original allo-HSCT, routinely prepared and stored under Good Manufacturing Practice conditions, retain the ability to induce GvL effects [2]. This proactive DLI strategy is applicable to graft preparations from granulocyte-colony stimulating factor (G-CSF)–primed and unprimed donors [3, 4].

The clinical outcome of allo-HSCT with optional DLI has been examined across a number of hematological cancer indications, as described in recent reviews [5–7]. Direct comparison of study results is hampered by the diversity of single-indication and mixed-indication basket designs used, with marked differences in patient selection and treatment settings. Thus, there is limited systematic information regarding allo-HSCT and DLI use patterns and outcome directly related to adult patients with AML. As a step toward future harmonized treatment strategies, a proposal for individualized, risk-adjusted use of DLI in AML patients has been published by the Acute Leukemia Working Party of the European Society for Blood and Marrow Transplantation [7].

The present study was designed to provide an update on our clinical experience by (i) focusing on AML in adults only; (ii) selecting a cohort that reflects the changes in the eligible patient population, notably older patients and those with comorbidities and extensive pretreatment, made possible by improved supportive care, inclusion of mismatched and unrelated donors, and a shift from myeloablative (MAC) toward reduced-intensity conditioning (RIC) [8]; and (iii) using a cohort with extensive follow-up that includes allo-HSCT with and without DLI from a single transplant center.

## Patients, materials, and methods

### Inclusion criteria and data collection

The electronic files of our unit at the Medical Center of the University of Freiburg, Germany, were used to identify all consecutive cases of allo-HSCT carried out between January 1, 2009, and December 31, 2017, in patients with confirmed diagnosis of AML and age ≥18 years. All patients had given their written informed consent to the treatment and use of information for research purposes. All analyses of human data were carried out in compliance with the relevant ethical regulations. All demographic data, treatment details, and information from regular clinical follow-up were prospectively collected in our dedicated allo-HSCT registry, with database lock for follow-up November 8, 2019, and analyzed retrospectively.

### Allo-HSCT and DLI procedures

Indication setting, diagnostic procedures, and clinical protocols followed international guidelines and institutional policies. Allo-HSCT were carried out with allogeneic peripheral blood hematopoietic stem cells (PBHSC), collected after G-CSF stimulation and prepared under Good Manufacturing Practice conditions. HLA-identical donors were classified as *HLA-matched* sibling or unrelated donors, and non-identical donors (e.g., HLA-A, B, C, DRB1, DQB1: 9/10) as *HLA-mismatched* sibling or unrelated donors, respectively. Initiation of DLI treatment, for which no specific AML guidelines have been put forward [8], followed individualized assessment of clinical status after transplantation, discontinuation of prophylactic immunosuppression, history and current status of GvHD (no active GvHD), eligibility for other treatment options, laboratory and clinical risk factors, and patient consent. Frozen aliquots of the original G-CSF stimulated graft were routinely stored for future use in DLI [2]. After using up the original aliquots, 5 patients with extended DLI dosing received further DLI with unstimulated donor lymphocytes from the original donor, a sibling, or an unrelated second donor, respectively. The selected DLI dose regimens were consistent with previous reports and recommendations that emphasize individualized dose adjustments [2, 7, 9]. Institutional policies did not include any a priori upper limits on the number of DLI cycles that could be given or the duration of DLI treatment.

We also considered the fact that clinical decisions to perform DLI influence the choice of additional treatment modalities [5], including various chemotherapy regimens (CT), hypomethylating agents (HMA: azacytidine, decitabine), and FLT3-directed tyrosine-kinase inhibitors (TKI: sorafenib, midostaurin). The corresponding information was collected for all patients with DLI.

### Genetic markers

Mixed chimerism in bone marrow or peripheral blood samples was assessed by fluorescence in situ hybridization (FISH) or by short tandem repeat (STR) analysis [10, 11]. For clinical assessment, mixed chimerism levels, with a conventional cut-off value of 5%, and kinetic profiles were considered risk indicators [7, 11]. The presence of leukemia cells in bone marrow or peripheral blood mononuclear cell preparations was defined by detection of specific mutations, rearrangements, or expression profiles for leukemia-associated genes (e.g., NPM1, FLT3, WT1, MLL, DEK, NUP214, CEBPA, CBFB, MYH11, RUNX1, RUNX1T1, EVI1, ETV6, AF6, AF9, AF10, RAEB-1, KMT2A, MLL3, PTD, PML-RARA, BCR-ABL, KIT, JAK2, TP53) [11] as part of the clinical laboratory practice at the Medical Center of the University of Freiburg, with continual updates of methods and procedures. The timing of mixed chimerism and genetic analyses was determined by routine clinical follow-up schedules, including bone marrow and peripheral blood analysis on day +30 post-transplant for remission assessment. Genetic data sets obtained with peripheral blood and bone marrow samples at the time of first diagnosis of AML in a given patient, when available, were aligned with the current version of the European Leukemia Network risk classification scheme (ELN 2017) [8].

### Response criteria and evaluation

The assessment of patient status followed international guidelines [8]. Briefly, CR was defined as bone marrow blasts < 5%, absence of circulating blasts and blasts with Auer rods, absence of extramedullary disease, absolute neutrophil count (ANC) ≥ 1000/μL, platelet count ≥ 100,000/μL, and transfusion independence. For the present study, we also included CR with incomplete hematological recovery in this category. Primary induction failure (PIF) was defined as failure to achieve any CR at any time despite treatment for AML. Progressive disease was defined as an increase in bone marrow blast percentage, increase of absolute blast counts in the blood, or new extramedullary disease. Relapse was defined as the recurrence of disease after CR, meeting one or more of the following criteria: ≥ 5% blasts in bone marrow or peripheral blood, extramedullary disease, or disease presence determined by clinical assessment. The temporal sequence of recurring remission and relapse events in a given patient, starting at time of AML diagnosis, was identified by sequential numbering (e.g., CR1, CR2, REL1).

The presence of aGvHD was recorded with overall grade (0=none; grades I–IV) and organ-specific stages for skin, liver, and intestine (0=none; stages 1–4) using standard criteria [12]. The presence of cGvHD was assessed by organ-specific scores (1 to 3) and global assessment of severity (mild, moderate, severe) [13], with updates [14]. Subcategories of aGvHD and cGvHD, including overlap syndromes [13], were not evaluated separately in the present study.

### Data analysis, endpoints, and statistics

The retrospective analysis of our single-center allo-HSCT database for adult AML patients included three hierarchically ordered steps for mutually exclusive and collectively exhaustive cohort partitioning (Online Resource 1a): (i) selection of first-time allo-HSCT only, since re-transplantation in AML is associated with a very different outcome [15]; (ii) selection of allo-HSCT meeting a combined landmark of CR on day +30 after transplantation and being alive without re-transplantation on day +100 vs those not meeting this landmark; and (iii) stratification of allo-HSCT meeting the combined day-100 landmark by DLI use, distinguishing between first-dose DLI given prior to any hematological relapse (preDLI), first-dose DLI given after relapse (relDLI), and no DLI during follow-up.

Patient-, disease-, and treatment-related variables of cohort subgroups were compared using Fisher’s exact test for categorical variables and the Mann–Whitney test for continuous variables. Baseline characteristics were summarized using median, interquartile range, and range for continuous measures and numbers and frequencies for categorical measures. As principal health outcome, we used survival with transplant [16, 17], defined here by the time from date of first allo-HSCT to death, from any cause, or date of re-transplantation, whichever occurred first, censored for status alive without re-transplantation at last follow-up. Overall survival, from date of first allo-HSCT to death, from any cause, censored for status alive at last follow-up, which aggregates benefit from first allo-HSCT and potential re-transplantation, was used for some comparative analyses. Conditional survival with first transplant was assessed with the day-100 landmark as described [18]. Follow-up times were assessed as described [19], accounting for death and re-transplantation. Probability of survival was estimated by the Kaplan–Meier method. Survival curves were compared by log-rank testing, and results were expressed as hazard ratios (HR) with 95% confidence intervals (95% CI). Cox proportional hazards regression models were used to assess impact of selected covariates at time of transplantation. Data were analyzed using GraphPad Prism version 8.3.0 (GraphPad Software, San Diego, CA, USA), accepting *p*<0.05 as indicating a statistically significant difference.

## Results

### Patient characteristics and GvL-directed therapy

A total of 342 adult patients with AML were treated with a first allo-HSCT at our unit between January 1, 2009, and December 31, 2017. The median age was 57 years (range 19–79), and the status at transplantation was active disease in 58% vs 42% with CR (Table 1). Toxicity-reduced conditioning was used in a majority of patients (79%), and graft donor selection showed a substantial contribution of unrelated HLA-matched (56%) and mismatched donors (22%). The median follow-up at time of database lock was 5.1 years.

**Table 1:** Clinical characteristics at time of first allo-HSCT

Patients	Total cohort	Day-100 landmark cohort	Day-100 landmark and DLI		
			no DLI	preDLI	relDLI
Number	342	292	199	42	51
Age [median (range)]	57 (19-79)	57 (19-78)	57 (19-78)	57 (32-75)	56 (22-78)
AML type					
De novo	235 *(69%)*	205 *(70%)*	135 (*68%*)	34 *(81%)*	36 *(71%)*
sAML/tAML^a^	107 *(31%)*	87 *(30%)*	64 (32%)	8 *(29%)*	15 *(29%)*
Status pre-HSCT					
Complete remission	145 *(42%)*	134 *(46%)*	95 (48%)	20 *(48%)*	19 *(37%)*
*CR1*	*122*	*111*	*79*	*17*	*15*
*CR2 or higher*	*23*	*23*	*16*	*3*	*4*
Active disease	197 *(58%)*	158 *(54%)*	104 (*52%*)	22 *(52%)*	32 *(63%)*
*PIF*	*109*	*84*	*56*	*9*	*19*
*REL1*	*40*	*33*	*20*	*6*	*7*
*REL2 or higher*	*2*	*2*	*1*	*1*	*-*
*Progression*	*5*	*3*	*3*	*-*	*-*
*Untreated*^*b*^	*41*	*36*	*24*	*6*	*6*
Conditioning					
Myeloablative	85 *(21%)*	75 *(26%)*	47 (24%)	12 *(29%)*	16 *(31%)*
*BU/CY-based*	*54*	*48*	*26*	*8*	*14*
*TT/BU/FLU*	*31*	*27*	*21*	*4*	*2*
Toxicity-reduced	257 *(79%)*	217 *(74%)*	152 (76%)	30 *(71%)*	35 *(69%)*
*FLU/BCNU/MEL*	*181*	*153*	*102*	*24*	*27*
*FLU/TT-based*	*68*	*56*	*50*	*3*	*3*
*Other FLU or TT*	*8*	*8*	*-*	*3*	*5*
HLA donor-to-recipient					
Unrelated matched	191 *(56%)*	164 *(56%)*	112 (56%)	23 *(55%)*	29 *(57%)*
Unrelated mismatched	70 *(20%)*	59 *(20%)*	37 (19%)	7 *(17%)*	15 *(29%)*
Sibling matched	72 *(21%)*	62 *(21%)*	44 (22%)	11 *(26%)*	7 *(14%)*
Sibling mismatched	4 *(1%)*	3 *(1%)*	2 (1%)	1 *(2%)*	-
Others^c^	5 *(1.5%)*	4 *(1.4%)*	4 (*2%*)	-	-

^a^Abbreviations: *sAML/tAML* secondary or therapy-related AML, *CR* complete remission, *PIF* primary induction failure, *REL* relapse, *BU* busulfan, *CY* cyclophosphamide, *FLU* fludarabine, *BCNU* carmustine, *MEL* melphalan, *TT* thiotepa. ^b^Up-front allo-HSCT predominantly in patients with sAML/tAML (32 of 41, total cohort; 29 of 36, day-100 landmark cohort; 12 of 12, DLI cohorts). ^c^Identical twins (2); haploidentical (2) or HLA-matched (1) child

Of the primary study cohort with first allo-HSCT, 292 of 342 patients (85%) met the combined day-100 landmark (CR on day +30; being alive without re-transplant on day +100) as the clinical setting most likely to qualify for future DLI use (Online Resource 1a). During follow-up, 93 patients (32%) received DLI, based on their clinical status, laboratory findings, availability of cryopreserved graft cells, and GvHD status. The first dose of DLI was given “preemptively”—prior to detecting any hematological relapse—in 42 patients (preDLI), based on routine monitoring for mixed chimerism and genetic markers combined with individual clinical risk assessment. Specifically, at the time of first-dose preDLI, 18 of 42 patients showed at least one molecular or cytogenetic risk marker, including 12 with molecular detection of target gene mutations, copy number changes, or increased expression, 5 with molecular genetic markers plus mixed chimerism, and one with cytogenetic changes plus mixed chimerism. Twenty-two patients showed mixed chimerism without reported genetic markers, and two had no reported genetic markers or mixed chimerism at the time of first preDLI dose. In addition, 51 patients received a first dose of DLI only after hematological relapse was detected (relDLI). As shown in Table 1, the clinical characteristics at time of transplantation are closely similar among total cohort, day-100 landmark cohort, and preDLI and relDLI cohorts, with no statistically significant differences identified.

### Characteristics of DLI use and additional treatment

**Fig. 1:**
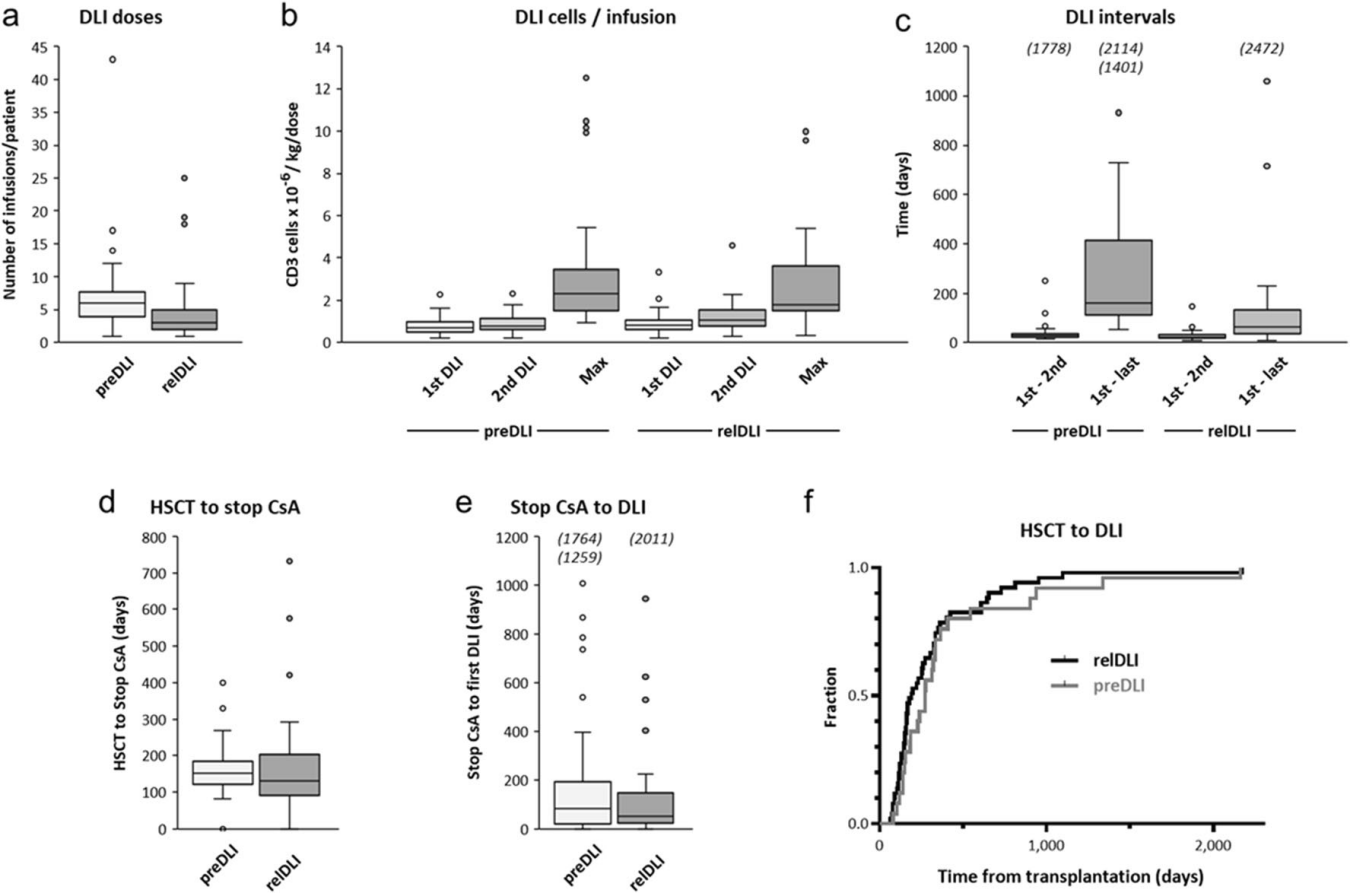
Characteristics of preDLI and relDLI dose regimens in adult AML patients with first allo-HSCT. Box-and-whisker plots for numbers of DLI doses/patient given during the study period, comparing preDLI and relDLI (a); numbers of CD3 cells/kg body weight given per DLI infusion, separately for first, second and, if applicable, third or later maximum individual dose (b); time intervals between first and second and, if applicable, first and last dose of DLI in a given patient (c); time intervals from transplantation to cessation of prophylactic immunosuppression per patient (d); and time intervals from cessation of prophylactic immunosuppression to first dose DLI per patient (e). In panel f, the preDLI and relDLI cohorts are compared for time delay between transplantation and first dose DLI. Data in panels a–c are right-censored for patients alive without re-transplantation at the time of last follow-up. In panels a–e, boxes represent 1st quartile, median and 3rd quartile values, whiskers extend by 1.5 times interquartile range beyond the 1st and 3rd quartiles, and outliers are represented by circles (or numbers in parentheses, in panel e)

The characteristics of DLI use in first-time allo-HSCT patients meeting the day-100 landmark (Table 2) were closely similar between preDLI and relDLI groups, and the only statistically significant differences seen were related to DLI dose regimens. Thus, the preDLI group showed a higher number of DLI cycles per patient than the relDLI group [median (range), 6 (1–43) vs 3 (1–25); *p*=0.0002), and, for patients with ≥ 2 cycles, a longer interval from first-dose to last-dose DLI [median (range), 161 days (53–2114) vs 65 days (8-2472); *p*<0.0001] (Fig. 1 a, c). The proportion of patients with ≥3 DLI cycles was 86% for the preDLI group vs 61% for the relDLI group. There was no statistically significant difference in the number cells per DLI between the preDLI and relDLI groups when comparing the respective first, second, and maximum doses given (Fig. 1 b). Regarding the temporal sequence, the median time from transplantation to discontinuation of prophylactic CsA, median time from discontinuing CsA to first-dose DLI, and median time from transplantation to first-dose DLI were comparable for the preDLI and relDLI groups (Fig. 1 d–f). Specifically, the time from transplantation to first-dose DLI was not statistically different in the preDLI and relDLI groups (median, 273 vs 191 days; *p*=0.13), or in the subset of preDLI-treated patients with subsequent hematological relapse (*n*=25) vs the relDLI group (median, 272 vs 191 days; *p*=0.37).

**Table 2:** Characteristics of allo-HSCT with DLI

Patients	Day-100 landmark cohort with DLI use	
	preDLI	relDLI
Allo-HSCT [number of patients]	42	51
Graft source: PBHSC fresh / cryopreserved	40 / 2	50 / 1
Donor sex: male / female	22 / 20	30 / 21
Donor age: median (range)	39 (20–68)	33 (19–69)
Graft cell numbers: median (range)		
*WBC × 10*^*−8*^*/kg BW*	*10.9 (4.2–22.9)*	*9.4 (5.2–29.9)*
*CD3+ cells × 10*^*−6*^*/kg BW*	*2.3 (0.1–6.8)*	*2.2 (0.1–6.4)*
*CD34+ cells × 10*^*−6*^*/kg BW*	*6.9 (2.4–21.0)*	*6.5 (1.9–17.0)*
Post-HSCT GvHD prophylaxis		
CSA/CAMPATH	25 *(60%)*	21 *(41%)*
CSA/MMF or MTX/ ± ATG	17 *(40%)*	30 *(59%)*
GvHD induced by HSCT prior to DLI		
Acute GvHD	14 *(33%)*	22 *(43%)*
*Grade: 1 / 2 / 3 / 4*	*11 / 3 / - / -*	*10 / 7 / 4 / 1*
Chronic GvHD (any grade)	8 *(19%)*	10 *(20%)*
DLI timing and dose regimen		
HSCT to DLI [days]: median (range)	273 (80–2164)	191 (63–2172)
Stop CSA to DLI [days]: median (range)	96 (8–1764)	52 (4–2011)
Total number DLI cycles/patient: median (range)	6 (1–43)	3 (1–25)
*n = 1*	*4 (10%)*	*9 (18%)*
*n = 2*	*2 (5%)*	*11 (22%)*
*n ≥ 3*	*36 (86%)*	*31 (61%)*
CD3+ cell dose [× 10^-6^/kg BW]: median (range)		
*First DLI*	*0.69 (0.20–2.27)*	*0.84 (0.22–3.56)*
*Second DLI*	*0.78 (0.20–2.31)*	*1.08 (0.31–4.59)*
*Third or later DLI (highest dose)*	*2.31 (0.94–12.51)*	*1.80 (0.34–10.02)*
DLI intervals (≥ 2 cycles) [days]: median (range)		
*First to second DLI*	*28 (14–1778)*	*22 (7–147)*
*First to last DLI*	*161 (53–2114)*	*65 (8–2472)*
GvHD following DLI		
Acute GvHD	12 *(29%)*	9 *(18%)*
Grade: 1 / 2 / 3 / 4	4 / 5 / - / 3	4 / 2 / 3 / -
Chronic GvHD (any grade)	6 *(14%)*	5 *(10%)*

*BW* body weight*, CSA* cyclosporin A*, MTX* methotrexate*, CAMPATH* alemtuzumab*, MMF* mycophenolate mofetil*, ATG* anti-thymocyte globulin

The incidence and severity of GvHD was monitored separately for the initial period after allo-HSCT, prior to first-dose DLI, and following DLI. The observed patterns for patients with preDLI and relDLI were not statistically different, as summarized in Table 2.

We also determined how commonly other treatment modalities, notably CT, HMA (azacytidine, decitabine), and FLT3-directed TKI (sorafenib, midostaurin), were introduced before or after a first dose of DLI. In the preDLI group (*n*=42), these added treatments were uncommon prior to first-dose DLI (TKI, 7% of patients; HMA, 5%), with a marked increase only when relapse occurred despite preDLI [subgroup with relapse (*n*=25): TKI, 24%; HMA, 52%; CT, 52%; HMA or CT, 80%]. In the relDLI group (*n*=51), the use of these treatments showed a similar pattern once relapse had occurred [TKI, *n*=13 (25%); HMA, *n*=32 (63%); CT, *n*=23 (45%); HMA or CT, *n*=44 (86%)], with the added treatment started either before or after patients received their first dose of relDLI in similar proportions: TKI start before/after first-dose relDLI in 5 vs 8 patients; HMA, 17 vs 15; CT, 11 vs 12; and HMA or CT, 26 vs 18.

### Outcome

**Fig. 2:**
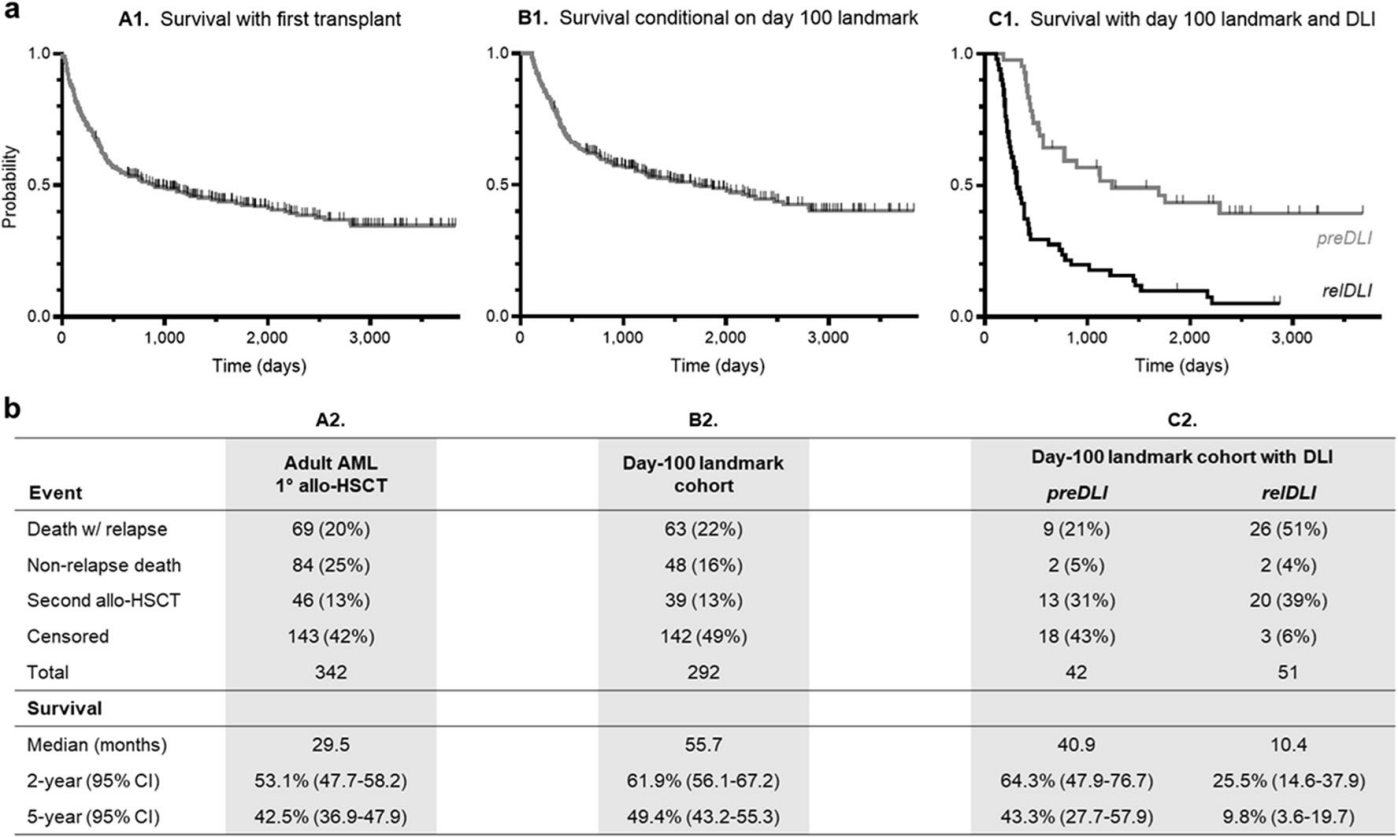
Survival with first allo-HSCT in adult patients with AML. Composite view of Kaplan–Meier plots (a) and tabulated outcome parameters (b), matched for the entire study cohort (graph A1, column A2), the day 100 landmark cohort (graph B1, column B2), and the day 100 landmark subcohorts with preDLI and relDLI (graph C1, column C2), respectively. Survival times from day of transplantation. In the top panel, vertical tick marks along the survival curves indicate individual patients alive without re-transplantation at the respective time of last follow-up. The bottom panel includes types of observed endpoint events for each group

Survival with first transplant was selected as principal health outcome. For the total cohort of first-time allo-HSCT (*n*=342), median survival was 29.5 months, with 2-year and 5-year estimates of 53% and 43%, respectively. In patients meeting the day-100 landmark (*n*=292; Fig. 2), the median survival was 55.7 months (2-year/5-year: 62%/49%). As expected, failure to meet the day-100 landmark, indicative of very early treatment-related mortality or rapid leukemia progression, was associated with low median survival (1.7 months) and a 2-year estimated survival of 2%.

Analysis of allo-HSCT that had met the day-100 landmark based on the type of DLI use revealed distinct outcomes (Fig. 2). Patients with preDLI (*n*=42) showed a median survival of 40.9 months (2-year/5-year: 64%/43%), and the observed endpoint events included relapse-related deaths (9 of 42; 21%), treatment-related deaths due to infection and GvHD (2 of 42; 5%), and re-transplantations (13 of 42; 31%), with 43% (18 of 42) alive without re-transplantation at last follow-up. In the relDLI setting (*n*=51), median survival was 10.4 months (2-year/5-year: 26%/10%). The observed endpoint events were relapse-related deaths (26 of 51; 51%), treatment-related death due to GvHD and infection subsequent to remission (1 of 51), death of unknown cause (1 of 51), and re-transplantation (20 of 51; 39%), with 6% (3 of 51) being alive without re-transplantation at last follow-up. The incremental benefit of re-transplantation in this setting, which was not the focus of our current study, is shown in Online Resource 2.

**Fig. 3:**
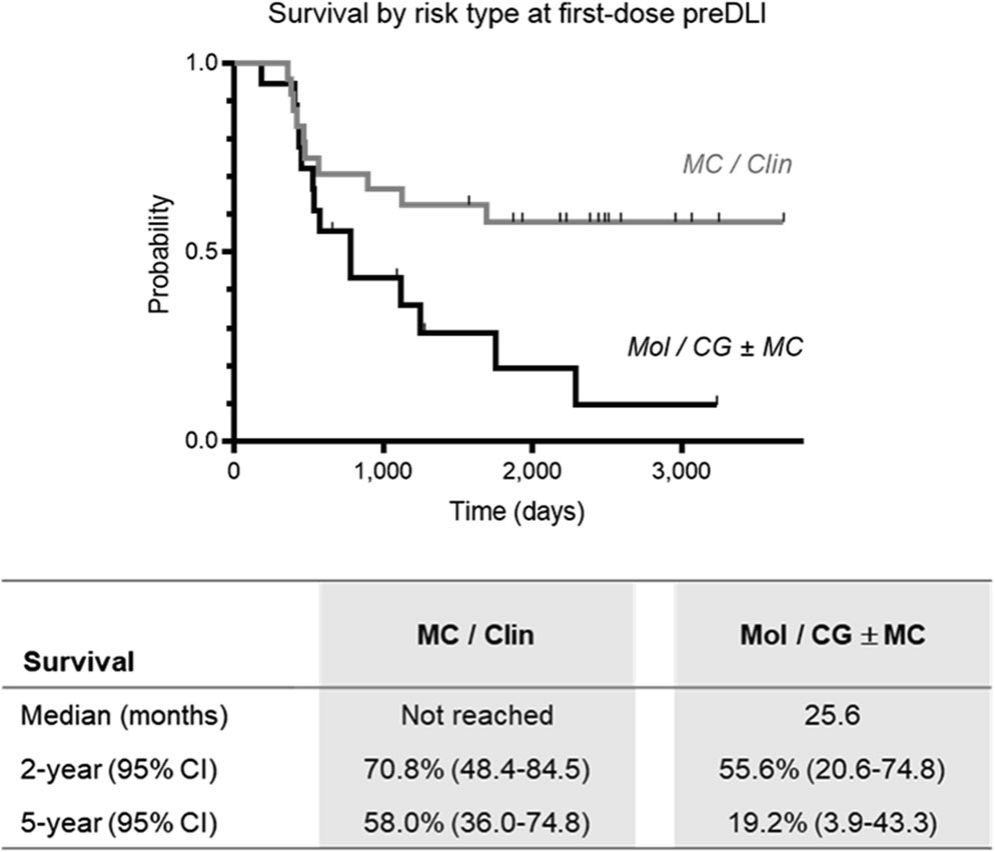
Survival with first allo-HSCT and preDLI by risk marker constellation at the time of first-dose preDLI. Kaplan–Meier plot and tabulated outcome parameters for the day 100 landmark cohort with preDLI use, stratified as follows: MC/Clin (*n*=24), includes 22 patients with detection of mixed chimerism (MC) and 2 with individual clinical risk assessment (Clin) at follow-up immediately preceding first-dose preDLI, without genetic risk markers at that time (*non-specific risk indicators*); Mol/CG ± MC (*n*=18), includes 17 patients with AML-typical molecular genetic findings (Mol), including 5 with concomitant mixed chimerism, and one with cytogenetic findings (CG), with concomitant mixed chimerism, reported at follow-up immediately preceding first-dose preDLI (*leukemia-specific risk indicators*). Survival times from day of transplantation

We further compared patients in the preDLI group who, just prior to receiving the first dose of DLI, were recorded either (i) with at least one of the more leukemia cell-specific genetic risk indicators (AML-typical gene mutations or expression patterns, or cytogenetic abnormalities), or (ii) only with less AML cell-specific risk indicators (mixed chimerism levels or kinetics; individualized physician’s judgement of clinical risk). As shown in Fig. 3, the subgroup with at least one genetic indicator (*n*=18) showed a median survival of 25.6 months (2-year/5-year: 56%/19.2%), and those without genetic indicator (*n*=24) did not reach median survival (2-year/5-year: 71%/58.0%). This difference was statistically significant [*p*=0.018; HR, 2.54 (95% CI, 1.10–5.86)].

For patients with no DLI during follow-up (*n*=199), median survival was not reached (2-year/5-year: 71%/62%). However, this group comprises two distinct clinical scenarios with very different prognosis and endpoint events. Thus, one subset of patients (37 of 199) relapsed after transplantation, received no DLI due to rapid progression or other treatment, and showed a low median survival of 8.4 months (2-year/5-year: 19%/7%). The observed endpoint events were relapse-related death (29 of 37; 78%), re-transplantation (6 of 37; 16%), unknown cause of death (1 of 37), or being alive without re-transplantation (1 of 37). The second subset with no DLI comprised patients that maintain CR (162 of 199) but remained at risk of delayed treatment-related mortality. In this setting, median survival was not reached (2-year/5-year: 83%/75%), 120 of 162 patients were alive at database lock, and 42 patients were reported with either treatment-related (38 of 42) or unknown (4 of 42) causes of death. The treatment-related causes (allowing multiple entries per patient) included GvHD in 14 of 38 patients; viral, bacterial, and fungal infections (21 of 38); post-transplant lymphoproliferative disorder (6 of 38); acute respiratory distress syndrome (3 of 38); and various types of single or multiple organ failure (10 of 38). As a cautionary note, this group with no DLI at database lock likely includes a proportion of patients that may receive DLI during their further clinical course, affecting our final outcome estimates. However, the median follow-up of 5.1 years achieved in this study (estimated 2-year follow-up: 95.2%; 95% CI 91.0–97), compared to the finding that most DLI are started within the first 2 years after allo-HSCT (Fig. 1 f), suggests that the necessary adjustments for DLI-based stratification, once follow-up is fully completed, should be moderate.

In a supplementary analysis, we examined all patients in the DLI-related subgroups who presented with hematological relapse during follow-up for the time from transplantation to relapse. The median time from transplantation to relapse was shorter in the relDLI subgroup (*n*=51; median 5.2 months) than the preDLI subgroup with relapse [*n*=25; 14 months; HR, 2.11 (95% CI, 1.34–3.3); *p*=0.0013]. In the no-DLI subgroup with relapse (*n*=37), median time to relapse was 5.4 months, similar to the relDLI subgroup. With regard to post-relapse survival with transplant, all three subgroups were closely similar (relDLI, median 3.7 months; preDLI, 3.5 months; no-DLI, 1.7 months).

Finally, we examined the correlation between disease status at the start of transplantation and outcome. As shown in Online Resource 3a, active disease vs CR at transplantation was associated with lower median survival in patients meeting the day-100 landmark [*p*=0.0017; HR, 1.68 (95% CI, 1.22–2.31)], with an estimated 5-year survival of 40.5% (95% CI 32.1–48.8) vs 59.3% (50.1–67.4). By contrast, no significant difference was seen for the preDLI and relDLI subgroups (Online Resource 3b-c). Instead, this difference can be traced to patients with continued CR and no DLI during follow-up [*p*=0.0059; HR, 2.38 (95% CI, 1.30–4.37)], the subgroup with mostly treatment-related mortality, with a 5-year estimated survival of 64.5% (95% CI 51.4–74.9) and 85.4% (75.1–91.7) for active disease status vs CR at the start of transplantation. Stratification of outcome according to the ELN 2017 classification was hampered by missing or partial data, notably for patients with early dates of initial AML diagnosis and no access to original diagnostic samples for re-analysis. Accepting these limitations, we assigned the DLI cohorts to favorable (preDLI: 7 of 37 evaluable; relDLI: 1 of 48 evaluable), intermediate (preDLI: 27 of 37; relDLI: 33 of 48), and adverse profiles at the time of initial AML diagnosis (preDLI: 3 of 37; relDLI: 14 of 48), respectively. With the caveat of small subgroup sizes, there was no statistically significant association between these risk categories and post-transplantation survival in either group.

## Discussion

The role of allo-HSCT in AML treatment depends on two key questions: which patients are treated with allo-HSCT—as a nonrandom selection step [20]—and what is the effectiveness of allo-HSCT thereafter? In our primary study cohort of patients with first allo-HSCT, the age distribution and representation of active disease at transplantation, secondary AML, and reduced-toxicity conditioning are consistent with the nonrandom selection of patients linked to broader eligibility for allo-HSCT [8]. This clinical profile is largely maintained throughout our subgroup analyses. As we start from a prospective single-center allo-HSCT database, we suggest that the estimated 2-year/5-year probability of survival of 53%/43% reflects the current level of effectiveness of first allo-HSCT in adult patients with AML. Moreover, the 2-year/5-year probability of survival is 62%/49% for patients meeting the day-100 landmark. This early landmark separates clinical courses with rapid leukemia progression or immediate treatment-related mortality [21], overwhelming any initial GvL benefit of the transplant, from those that offer extended options for further GvL-modifying strategies.

In our day-100 landmark cohort, DLI were included in the clinical management of about one-third of patients (32%). There are several general criteria to be met when deciding on DLI eligibility, notably the availability of suitable graft donor cells, acceptable GvHD levels after allo-HSCT, and lack of more promising treatment choices [7]. In addition, there is an option, based on the individualized clinical assessment of each patient, to implement a first dose of DLI “preemptively,” based on preceding clinical and laboratory risk markers, or to implement a first dose of DLI after detecting a relapse. In our day-100 landmark cohort, these two options were selected in about equal proportions of patients (14% vs 17%). The 2-year/5-year probabilities of survival with first transplant are 64%/43% for the preDLI cohort, 26%/10% for the relDLI cohort, and 71%/62% for the cohort with no DLI during follow-up. Within the preDLI cohort, patients who presented prior to first-dose DLI with leukemia-specific molecular or cytogenetic risk markers showed inferior survival compared to those with less specific risk indicators, namely mixed chimerism and/or individual clinical risk ranking. This difference was statistically significant but due to limited group sizes and the heterogeneity of detected gene markers in our cohort, larger follow-up studies will be important.

The specific criteria selected for our preDLI/relDLI classification, namely comparing the date of first-dose DLI to the date of any potential post-transplantation relapse, reflect the need for an objective, unambiguous, and retrospectively retrievable set of parameters in existing allo-HSCT databases to allow cohort partitioning among patients with highly diversified treatment patterns (Online Resource 1 a). In this respect, we diverge slightly from proposed classifications [22] that assume differences in *DLI intent* of treating overt hematological relapse, treating measurable residual disease (MRD) [23, 24], or preventing both MRD and relapse with prospectively scheduled adjuvant DLI [25–27]. Lessons learned from chronic myeloid leukemia [22] show that the intent-based classification requires a robust and universally applicable set of laboratory markers to distinguish MRD-positive and MRD-negative patients and to serve as reliable mechanistic biomarkers for short-term DLI effects. Establishing a similar framework for AML is highly desirable but, as outlined by Ravandi et al. [23], not yet achieved in light of the distinctive heterogeneity and clonal architecture of AML. Current limitations include the failure of existing technology platforms (i.e., AML gene mutations and expression, cytogenetics, genome typing, multi-parameter flow cytometry, mixed chimerism) to identify and standardize single-timepoint criteria for the MRD-positive status, and to distinguish between bona fide MRD-negative patients and AML without appropriate detection method; e.g., available targets for quantitative PCR may cover only about 50% of all AML cases [23]. Secondly, even specific gene markers may not distinguish leukemic cells that are biologically capable and likely to cause relapse from residual cells that are terminally differentiated or in senescence. Third, there is no randomized controlled trial evidence yet to recommend MRD monitoring in AML as a validated interim endpoint for patient benefit [23, 28, 29]. These limitations explain why previous studies in AML have differed markedly in their technical details to identify preemptive and prophylactic DLI use and select study cohorts, preventing direct comparison of the results [5–7]. For the purposes of our day-100 landmark design, which aims to exclude no patients, it is even more important that we define preDLI and relDLI in an objective and unambiguous manner. The limited ability to classify a total allo-HSCT cohort in AML by robust MRD-positive and MRD-negative status, as outlined above, also explains why our landmark design incorporates the genetic and non-specific risk factors at the level of the preDLI group only, and not as a first-tier diagnostic category (Online Resource 1 b).

Our study includes additional aspects that extend or diverge from previous reports of allo-HSCT with DLI in AML patients. For instance, the studies cited in recent reviews [5–7] do not yet provide a comprehensive cohort perspective for allo-HSCT, with and without DLI, or a linked analysis of preDLI and relDLI, to account for shifts in nonrandom patient selection and additional treatment modalities. In addition, few studies have concentrated on AML only. Instead, many have used basket designs, aggregating data for adult AML with, variously, pediatric AML, myelodysplastic syndrome, myeloproliferative neoplasm, acute lymphocytic leukemia, or other hematological conditions [2–4, 30–33]. Finally, exclusion criteria have been used widely but inconsistently to limit the analysis to specific clinical settings. Collectively, most of these studies advocate some form of DLI in AML [2–4, 15, 25, 27, 30, 33–39], but a uniform assessment of DLI effectiveness in adult AML has not yet emerged. The present study may help to fill some of these gaps specific to AML, and our day-100 landmark design is readily transferable to other existing allo-HSCT databases for direct comparison while retaining the contextual information of nonrandom patient selection, prevalence and type of DLI use, patterns of added modalities, and outcome.

There are several limitations to be considered. As a general caveat for studies in AML with allo-HSCT [28, 29], our findings do not establish specific efficacy claims for DLI that would require randomized controlled trials. It also deserves emphasis that our results do not predict how outcomes would change with different decision criteria for preDLI or relDLI use. Importantly, the no-DLI group is not an internal control for preDLI and relDLI decision making but, instead, describes two distinct event sequences: relapsed patients ineligible for relDLI or prioritized for re-transplantation, and patients with continued CR, but still facing the risk of late treatment-related mortality [21, 40]. Finally, it is inherent to our cohort design that some of the patients without DLI and censored at the time of database lock may still receive DLI during their subsequent clinical course. Since we and others [9, 36, 41] have found that most DLI decisions are taken within the first 2 years after allo-HSCT, it seems likely that these adjustments in final cohort assignment will decrease over time, and our median follow-up of 5.1 years should suffice to support our main study conclusions.

As an incentive to future research, we report an unexpected observation regarding the temporal alignment between date of allo-HSCT and dates of first-dose DLI and post-transplantation relapse, respectively. Previously, the benefit of preemptive DLI had been linked directly to an early-warning concept, with the assumption that a first dose of preemptive DLI would generally occur sooner after transplantation than a first dose of DLI triggered by hematological relapse [42]. However, previous studies have not examined this assumption in the context of uniform institutional policies for the clinical management of a common starting cohort of adult AML patients with allo-HSCT. We identified no statistically significant difference between median time from allo-HSCT to first-dose preDLI vs first-dose relDLI. While the preemptive DLI setting, by definition, describes an early (*prior-to-relapse*) intervention in a given patient, this may be counteracted at the cohort level. Conceivably, a priori differences among allo-HSCT recipients regarding clonal genetics of residual leukemic cells [43] or propensity for slow vs precipitous escape from GvL surveillance [44] may allow detection of an extended period of steady-state MRD in some patients, while the same monitoring schedule may fail to detect a discrete MRD phase in patients with a steep rise in leukemic cell burden. In this scenario, non-random selection of the preDLI and relDLI treatment options, reflecting as yet unidentified leukemic cell attributes, is superimposed on intrinsic DLI efficacy. Separating these elements will require dual progress in the efforts to establish MRD as a bona fide diagnostic entity in AML and to implement standardized MRD surveillance, as a justification for randomized controlled trials of routinely cryopreserved DLI in early phases after allo-HSCT.

## Supplementary Information

ESM 1 (PDF 1580 kb)

## Data Availability

The datasets generated during and/or analyzed during the current study are available from the corresponding author on reasonable request.

## Declarations

The research protocol was approved by the institutional ethics committee.
