# Supplementary material for: Donor lymphocyte infusions after first allogeneic hematopoietic stem-cell transplantation in adults with acute myeloid leukemia: a single-center landmark analysis
Source: Ann Hematol. 2021 Apr 1;100(9):2339–50. doi: 10.1007/s00277-021-04494-z (PMC8357755; doi:10.1007/s00277-021-04494-z)
Supplement: Supplementary file 1 — (PDF 1580 kb) [file 277_2021_4494_MOESM1_ESM.pdf]

Electronic Supplementary Material 1

Donor lymphocyte infusions after first allogeneic hematopoietic stem-cell transplantation in adults with acute myeloid leukemia: a single-center landmark analysis

Annals of Hematology

Andrés R. Rettig[1], Gabriele Ihorst[2], Hartmut Bertz[1], Michael Lübbert[1], Reinhard Marks[1], Miguel Waterhouse[1], Ralph Wäsch[1], Robert Zeiser[1], Justus Duyster[1], Jürgen Finke[1]

[1]Department of Hematology, Oncology and Stem Cell Transplantation, University Medical Center Freiburg, Freiburg, Germany

[2]Clinical Trials Unit, University Medical Center Freiburg, Freiburg, Germany

Corresponding author: Andrés R. Rettig; e-mail: andres.rettig@uniklinik-freiburg.de

Online Resource 1

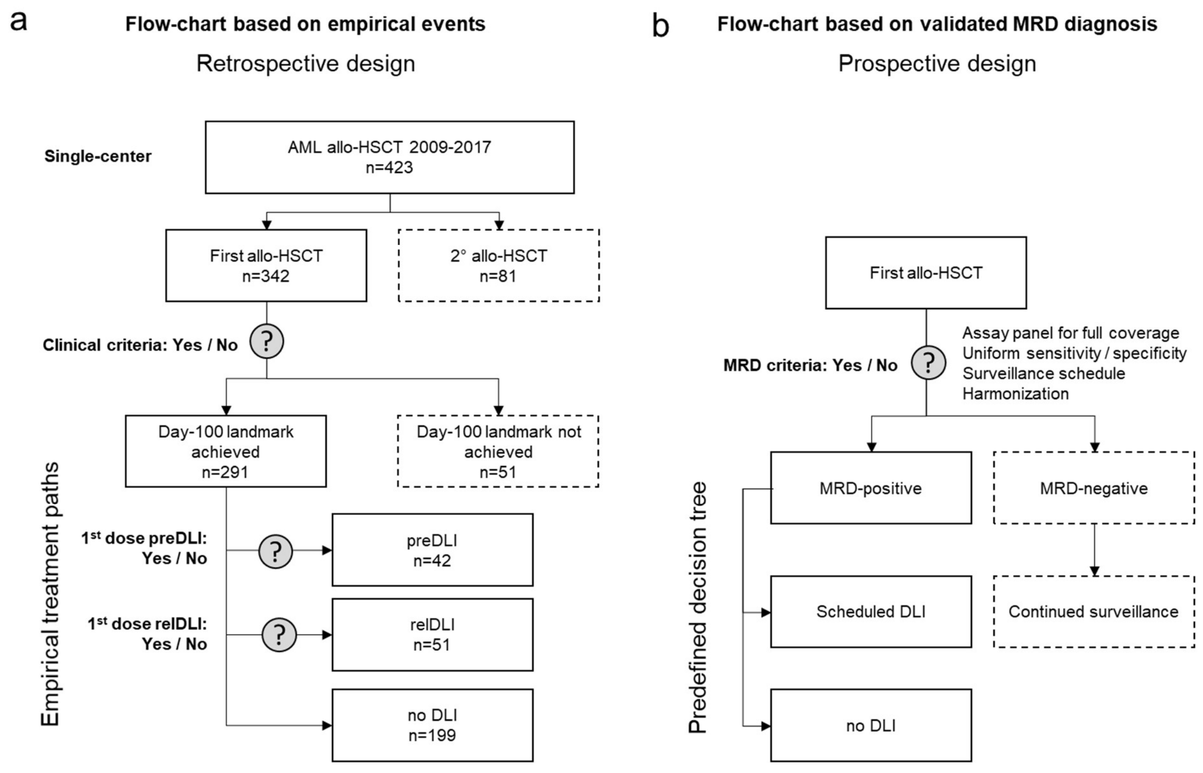

*Schematic representation of (**a**) cohort partitioning algorithm used in the present study, with achievement of day 100 landmark criteria (Yes / No) and documented date of first-dose DLI treatment, either before (preDLI, Yes / No) or after date of first detection of hematological relapse (relDLI, Yes / No) as mutually exclusive principal events. Part (**b**) depicts a hypothetical future scenario in AML with allo-HSCT, in which a standardized, sensitive and specific clinical entity of Measurable Residual Disease (MRD) becomes available as an approach to principal cohort stratification (cf. Ravandi et al., Blood Adv 2:1356–1366, 2018).*

Online Resource 2 Comparison of outcome results for adult AML patients with first allo-HSCT based on survival-with-transplant vs conventional overall survival including re-transplantation

a

Survival after first allo-HSCT: endpoint comparison

Endpoint	Adult AML 1° allo-HSCT	Day-100 landmark cohort	Day-100 landmark cohort and DLI		
			no DLI	*preDLI*	*relDLI*
**Survival-with-transplant [a]**					
Median (months)	29.5	55.7	Not reached	40.9	10.4
2-year (95% CI)	53.1% (47.7-58.2)	61.9% (56.1-67.2)	70.8 % (63.9-76.6)	64.3% (47.9-76.7)	25.5% (14.6-37.9)
5-year (95% CI)	42.5% (36.9-47.9)	49.4% (43.2-55.3)	62.4 % (54.9-69.0)	43.3% (27.7-57.9)	9.8% (3.6-19.7)
**Overall survival [b]**					
Median (months)	*36.9*	*65.8*	Not reached	*56.0*	*16.2*
2-year (95% CI)	57.5% (52.1-62.6)	65.7 % (59.9-70.8)	72.5 % (65.5-78.0)	71.3 % (55.0-82.6)	35.3 % (22.6-48.2)
5-year (95% CI)	44.7% (39.0-50.2)	51 % (57.3-46.1)	63.5 % (56.0-70.1)	46.6 % (30.6-61.6)	11.8 % (4.5-22.8)

[a] Survival with transplant defined by date of first transplantation to the earlier of death, from any cause, or re-transplantation, censored for status alive at last follow-up. [b] Overall survival defined by date of first transplantation to death, from any cause, censored for status alive at last follow-up.

b

Day-100 landmark cohort

Survival after first allo-HSCT by disease status at transplantation: endpoint comparison

Status at first allo-HSCT	Median (months)	2-year (95% CI)	5-year (95% CI)	P-value	HR Active disease vs CR	95% CI of ratio
**Survival-with-transplant [a]**						
CR [c]	Not reached	69.2% (60.6-76.2)	59.3% (50.1-67.4)			
				0.0017	1.68	(1.22-2.31)
Active disease	32.7	55.7% (47.6-63.0)	40.5% (32.1-48.8)			
**Overall survival [b]**						
CR	Not reached	74.5% (66.2-81.1)	62.2% (53-70.1)			
				0.0002	1.89	(1.36-2.62)
Active disease	36.8	58.8% (50.7-66.0)	41% (32.4-49.4)			

[a] Survival with transplant defined by date of first transplantation to the earlier of death, from any cause, or re-transplantation, censored for status alive at last follow-up. [b] Overall survival defined by date of first transplantation to death, from any cause, censored for status alive at last follow-up. [c] Disease status at transplantation: CR, complete remission vs. active disease

Online Resource 3 Covariate analysis for adult AML patients with first allo-HSCT based on disease status at transplantation

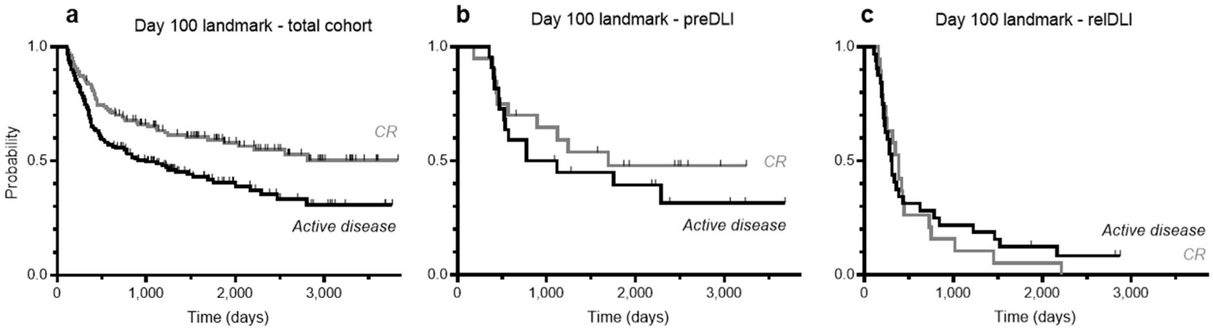

Cohort	Status at HSCT	Median survival (months)	HR	95% CI of ratio	P-value
Day-100 landmark - total	Active disease vs CR	32.7 vs not reached	1.68	1.22-2.31	0.0017
Landmark with preDLI	Active disease vs CR	31.2 vs 55.7	1.41	0.63-3.14	0.40
Landmark with relDLI	Active disease vs CR	12.6 vs 9.9	0.89	0.49-1.59	0.68

*Comparison of survival with first transplant in patients who were either in CR or had active disease at the time of transplantation. The top panels show Kaplan-Meier plots for all patients meeting the day 100 landmark (**a**) and subgroups meeting the day 100 landmark and receiving either preDLI (**b**) or relDLI (**c**). Survival times are shown from day of transplantation. Vertical tick marks along the survival curves indicate individual patients alive without re-transplantation at the respective time of last follow-up. The bottom panel summarizes the results of univariate comparisons for survival and pre-transplantation clinical status.*
